# Evaluation of kidney stone–related renal infection status by 18F-FDG PET/CT in lung cancer patients with concomitant nephrolithiasis: a multicenter study

**DOI:** 10.3389/fimmu.2026.1744163

**Published:** 2026-05-19

**Authors:** Xiangyang Yao, Xiaolong Ying, Hua Liu, Chen Duan, Bo Li, Zongyao Hao, Zhangqun Ye, Hua Xu, Hongbing Mei, Haoran Liu, Jianhe Liu

**Affiliations:** 1 Department of Urology, The Second Affiliated Hospital of Kunming Medical University, Kunming, China; 2 Department of Urology, Shenzhen Second People's Hospital, The First Affiliated Hospital of Shenzhen University, Shenzhen, China; 3 Department of Urology, The First Affiliated Hospital of Anhui Medical University, Hefei, China; 4 Department of Urology, Zhongnan Hospital of Wuhan University, Wuhan, China; 5 Department of Urology, Tongji Hospital, Tongji Medical College, Huazhong University of Science and Technology, Wuhan, China

**Keywords:** CaOx, kidney stone, nephrocalcinosis, PET/CT, renal infection

## Abstract

**Introduction:**

Positron emission tomography/computed tomography (PET/CT) has demonstrated significant clinical utility in the localization of infectious foci, yet its application in nephrolithiasis-associated renal infections remains underexplored.

**Methods:**

This retrospective cohort study assessed the diagnostic efficacy of ^18^F-FDG PET/CT in 120 lung cancer patients with radiologically confirmed nephrolithiasis across multiple centers. Quantitative analyses included calculation of diagnostic accuracy parameters, Pearson correlation between standardized uptake values (SUVmax), calculi burden, and serum cytokine profiles. Complementary experimental validation was performed using a murine calcium oxalate (CaOx) nephrocalcinosis model, utilizing micro-CT to analyze crystal deposition and inflammatory activity.

**Results:**

Clinically, PET/CT detected focal FDG uptake in 77 patients (64.2%), demonstrating 85.7% sensitivity and 76.7% specificity for infection identification. SUVmax positively correlated with stone burden and proinflammatory cytokines. The larger the size of the stone, the more severe the kidney infection, which in turn further aggravated the development of the stone. Furthermore, preclinical micro-PET/CT in CaOx mice revealed spatial concordance between crystal deposition and metabolic activity, paralleling human cytokine trends.

**Discussion:**

In lung cancer patients with concomitant nephrolithiasis, ^18^F-FDG PET/CT demonstrates high diagnostic yield for detecting renal infection foci and may serve as a valuable tool for preoperative risk assessment. These findings provide a foundation for future studies in broader nephrolithiasis populations.

## Introduction

Computed tomography (CT) and related imaging technologies have advanced quickly, greatly improving nephrolithiasis diagnostic capabilities (1, 2). In contrast to traditional radiography, CT achieves higher stone detection accuracy by overcoming the constraints caused by anatomical overlap and intestinal gas interference (3, 4). When integrated with three-dimensional (3D) reconstruction, CT further enables precise stone localization and generates intuitive anatomical visualizations, thereby informing surgical planning and therapeutic strategy selection (5–7). Nevertheless, despite these developments, conventional CT performs less well in detecting renal parenchymal infections brought on by nephrolithiasis or calcium oxalate (CaOx) nephrocalcinosis. This is especially true because of resolution limitations that make it difficult to detect encapsulated infections or early-stage inflammatory foci (8). This diagnostic flaw has significant therapeutic ramifications since undiagnosed infections can develop subtly and significantly raise the risk of postoperative sepsis in patients having percutaneous nephrolithotomy (PCNL).

Positron emission tomography/computed tomography (PET/CT) is a nuclear medicine imaging technique that integrates metabolic and anatomical data. The infusion of ^18^F-fluorodeoxyglucose (^18^F-FDG), a glucose analog that preferentially accumulates in cells with increased glycolytic activity, is what gives it its diagnostic value (9). This property has established PET/CT as a cornerstone in oncology for mapping malignant lesions (8–12), while emerging evidence supports its value in quantifying inflammatory burden during infectious processes (13–15). Mechanistically, even in the pre-abscess stages of infection, activated inflammatory cells (e.g., neutrophils, macrophages) at infection sites exhibit upregulated glucose transporter (GLUT) expression and hexokinase activity, leading to enhanced ^18^F-FDG avidity even during pre-abscess stages of infection (16–19). Notably, prior clinical observations have validated PET/CT’s capability to detect infected renal cysts in polycystic kidney disease through characteristic metabolic patterns, suggesting its potential as an early diagnostic tool for renal infections (20–24). Nevertheless, critical knowledge gaps persist regarding its diagnostic performance in stone-associated pyelonephritis, especially when it comes to distinguishing metabolic signals from sterile inflammation caused by CaOx crystals from actual microbial infections.

This diagnostic uncertainty carries particular clinical significance given the insidious nature of nephrolithiasis-related infections. Asymptomatic calculi may progress to pyelonephritis or renal failure despite unremarkable routine laboratory findings (27, 28), while occult infections in PCNL candidates heighten perioperative sepsis risk. Current diagnostic paradigms lack the sensitivity to reliably identify these clinically silent infections, underscoring the need for advanced metabolic imaging approaches.

In this multicenter retrospective study, we investigated the diagnostic efficacy of ^18^F-FDG PET/CT in two complementary models, including lung cancer patients with concomitant nephrolithiasis undergoing preoperative PET/CT staging, and a murine model of CaOx-induced nephrocalcinosis. Our dual approach combines clinical correlation with mechanistic insights, evaluating PET/CT’s potential for preoperative infection assessment and disease monitoring. To our knowledge, this represents the first comprehensive investigation of metabolic imaging’s role in managing stone-related renal infections in patients with lung cancer, providing a foundation for future studies in broader patient populations.

## Materials and methods

### Patient population and setting

This research project involves Tongji Hospital of Huazhong University of Science and Technology, the Second Affiliated Hospital of Kunming Medical University, and the First Affiliated Hospital of Anhui Medical University, spanning from January 2018 to January 2025. Because PET/CT imaging is routinely performed for oncologic staging and treatment monitoring in lung cancer, this population provided a clinically available cohort to retrospectively evaluate renal metabolic activity in patients with concomitant nephrolithiasis. Patients were diagnosed with unilateral kidney stone by imaging methods (KUB, ultrasound, or CT) and had undergone at least one PET/CT scan. Patients with stone >2cm, metabolic and autoimmune disease, ureteral stone and hydronephrosis (grade 2 or 3 according to The Society for Fetal Urology guidelines), and renal cancer were excluded. Large intrarenal stones (>2 cm) and ureteral stones were excluded because they may cause urinary obstruction or delayed ¹^18^F-FDG excretion, leading to tracer retention in the renal pelvis that can mimic or obscure true parenchymal infection, thereby confounding image interpretation. The local institutional review board approved this multicenter, retrospective study and the requirement for written informed consent (PJ-2020-137).

### Data collection

The hospital electronic case database was reviewed in detail, and data were collected for relevant clinical information (age, gender, kidney stone size, PET/CT imaging results, laboratory values (C-reactive protein, white blood cell count, blood cultured pathogen and serum cytokine levels), medical history, duration of hospital stay and follow-up data. Due to the retrospective design of this multicenter study, certain clinical details—such as precise antibiotic use prior to PET/CT, detailed symptomatology, and comprehensive urinalysis findings—were not consistently documented across all participating centers and thus were not included in the analysis. In addition, we retrospectively collected data on antibiotic use within 7 days prior to PET/CT (including duration), presenting symptoms (fever ≥38.0 °C, flank pain, lower urinary tract symptoms), urinalysis findings (microscopic white blood cell and red blood cell counts), and non-contrast CT features (hydronephrosis grade, perinephric stranding). These supplemental clinical characteristics are summarized in **Table 1**. A total of 120 patients were identified with renal stone based on their initial PET/CT examination. In addition, the uptake of ^18^F-FDG in renal parenchymal regions was evaluated in 18 patients with nephrolithiasis who underwent two PET/CT scans within a six-month interval.

**Table 1:** Clinical characteristics of 120 patients with kidney stones.

Characteristic	All patients (120)	PET/CT positive (77)	PET/CT negative (43)	*P* value
Antibiotic use within 7 days prior to PET/CT				0.21
Yes	43 (35.8%)	25 (32.5%)	18 (41.9%)	
No	77 (64.2%)	52 (67.5%)	25 (58.1%)	
Antibiotic duration (days), mean ± SD	5.2 ± 2.1	4.8 ± 1.9	5.7 ± 2.3	0.15
Symptoms	83.2 (63)			
Fever (≥38.0°C)	52 (43.3%)	41 (53.2%)	11 (25.6%)	0.003
Pain (stone side)	68 (56.7%)	50 (64.9%)	18 (41.9%)	0.01
Lower urinary tract symptoms	41 (34.2%)	29 (37.7%)	12 (27.9%)	0.28
Asymptomatic	23 (19.2%)	9 (11.7%)	14 (32.6%)	0.006
Urinalysis findings				
WBC, mean ± SD	12.4 ± 8.7	15.8 ± 9.2	6.3 ± 4.1	<0.001
RBC, mean ± SD	8.6 ± 6.3	9.1 ± 6.8	7.7 ± 5.4	0.24
Non-contrast CT findings				
Hydronephrosis	86 (71.7%)	51 (66.2%)	35 (81.4%)	
Perinephric stranding	22 (18.3%)	18 (23.4%)	4 (9.3%)	0.049

Lower urinary tract symptoms include frequency, urgency, and dysuria.

*WBC*, white blood cell; *RBC*, red blood cell; *HPF*, high-power field; *SD*, standard deviation.

### ^18^F-FDG PET/CT scanning and image interpretation

Following a standardized 6-hour fasting protocol, patients received intravenous ^18^F-FDG (4 MBq/kg) with PET/CT imaging (GE Discovery VCT XT, Milwaukee, WI, USA) performed 60 minutes post-injection. To minimize urinary retention artifacts, a threshold-based segmentation model (SUV <2.0) was applied to exclude renal pelvis radioactivity. Stone volume was quantified using semi-automated 3D segmentation with a threshold of over 100 Hounsfield units, followed by manual verification. Metabolic activity was evaluated by comparing the maximum standardized uptake value (SUVmax) between the affected and contralateral kidneys. Focal ^18^F-FDG uptake exceeding adjacent tissue activity, discordant with physiological biodistribution patterns and lacking pathognomonic features of non-infectious pathologies, was classified as infection-positive. Definitive diagnoses necessitated comprehensive validation by combining laboratory biomarkers, imaging-pathophysiological correlations, and monitoring of therapeutic responses. Two board-certified nuclear medicine physicians specializing in infection imaging conducted double-blind independent reviews. Discordant interpretations underwent structured multidisciplinary panel review, incorporating longitudinal clinical trajectory analysis. Final determinations followed evidence-based consensus adjudication.

### Composite reference standard

In the absence of a single definitive diagnostic test for stone-related renal infection, a composite reference standard (CRS) was used as the comparator. The CRS was defined by integrating microbiological results, clinical diagnosis, and follow-up findings. Specifically, renal infection was considered present when at least two of the following criteria were fulfilled:(1) positive urine culture obtained during hospitalization;(2) elevated inflammatory markers (e.g., leukocytosis or elevated C-reactive protein) with a discharge diagnosis of urinary tract infection or pyelonephritis;(3) clinical improvement after targeted antimicrobial therapy during follow-up (25, 26). Clinical follow-up was conducted for a minimum of six months after hospital discharge, with documentation of symptom resolution and/or negative follow-up urine cultures when available.

PET/CT findings were not incorporated into the reference standard for infection diagnosis. Clinical classification was determined prior to imaging analysis and served as the comparator for subsequent evaluation of PET/CT performance. In cases with discordant findings among individual criteria, final classification was adjudicated through multidisciplinary review by urologists and infectious disease specialists, based on comprehensive assessment of the clinical course and treatment response.

### Micro-CT SkyScan imaging

Recent studies highlight micro-computed tomography (micro-CT) as a transformative tool for investigating pathophysiological mechanisms and therapeutic responses in nephrolithiasis models, serving both preclinical research and clinical diagnostic enhancement (29, 30). In our experimental protocol, calcium oxalate (CaOx) nephrocalcinosis in murine kidneys was characterized using a high-resolution micro-CT system (SkyScan 1176, Bruker, Billerica, MA, USA) at 55 kVp with a 9µm isotropic voxel resolution. The total acquisition time for each kidney scan was approximately 30 minutes. Post-processing through CTVox 2.1 software enabled precise volumetric visualization of crystalline deposits, permitting three-dimensional quantification of total stone burden and spatial distribution patterns. This non-destructive technique demonstrated superior sensitivity in detecting submillimeter microcalculi compared to conventional histopathological methods.

### Animal experiment

In accordance with the “Guidelines for the Care and Use of Laboratory Animals of the National Institutes of Health”, 6- to 8-week-old C57BL/6J mice were approved by the Ethics Committee of Tongji Hospital and raised in a specific pathogen-free animal facility. The mice were randomly allocated into five groups (n=6/group) using a computer-generated sequence: Control group; Gly group; Gly+FICZ group; Gly+antagomiR-142 group; Gly+FICZ+antagomiR-142 group, drug dosage was referred to our previously published article. Each mouse in the control group was injected intraperitoneally with normal saline (100 µl) on seven consecutive days, while the treatment group was injected with glyoxylic acid (100 mg/kg) (27, 28). The dose of 6-formylindolo (3, 2-b) carbazole (FICZ) were intraperitoneally injected with from day 1 to day 10 (100 mg/kg); and the antagomiR-142 were caudal vein injected on days 1, 4, and 7 (20 mg/kg, 100 µl) (29, 30). Following a 12-h fast, mice underwent ¹^18^F-FDG PET/CT imaging (200 ± 10 μCi via tail vein) using a Trans-PET BioCaliburn 700 system (Leikan Technology, Wuhan, China) in static acquisition mode. Subsequently, AMIDE software was used to analyze interesting data. Images were evaluated by three nuclear medical physicians who were blinded to the clinical information. All mice were sacrificed in a CO_2_ box after 7 days, and kidney tissue was collected for fixation. This murine CaOx nephrocalcinosis model was used to evaluate crystal deposition-associated renal inflammatory responses and corresponding ^18^F-FDG uptake and was intended to reflect sterile crystal-induced renal inflammation rather than active microbial infection.

### Serum cytokines

Cytokine levels (IL-1β, TNF-α, IL-10, IL-6) were measured using commercial ELISA kits following standardized protocols. IL-1β (DY401), TNF-α (DY410), and IL-10 (DY417) assays were sourced from R&D Systems (USA), whereas IL-6 was measured using the BMS603–2 kit from Thermo Fisher Scientific (USA). All measurements were performed in technical duplicates with strict adherence to manufacturers’ quality control specifications.

### Statistical analysis

Biological data were processed using GraphPad Prism 7.0 and presented as mean ± standard deviation. Statistical significance between two groups was determined by Student’s t-test, whereas multiple-group comparisons were performed using one-way ANOVA followed by Tukey’s *post hoc* test. Bivariate correlations involving stone dimensions, cytokine profiles, and SUVmax values were assessed using Pearson’s correlation analysis. A p-value of < 0.05 was considered statistically significant.

## Results

### Patient population

**Figure 1:**
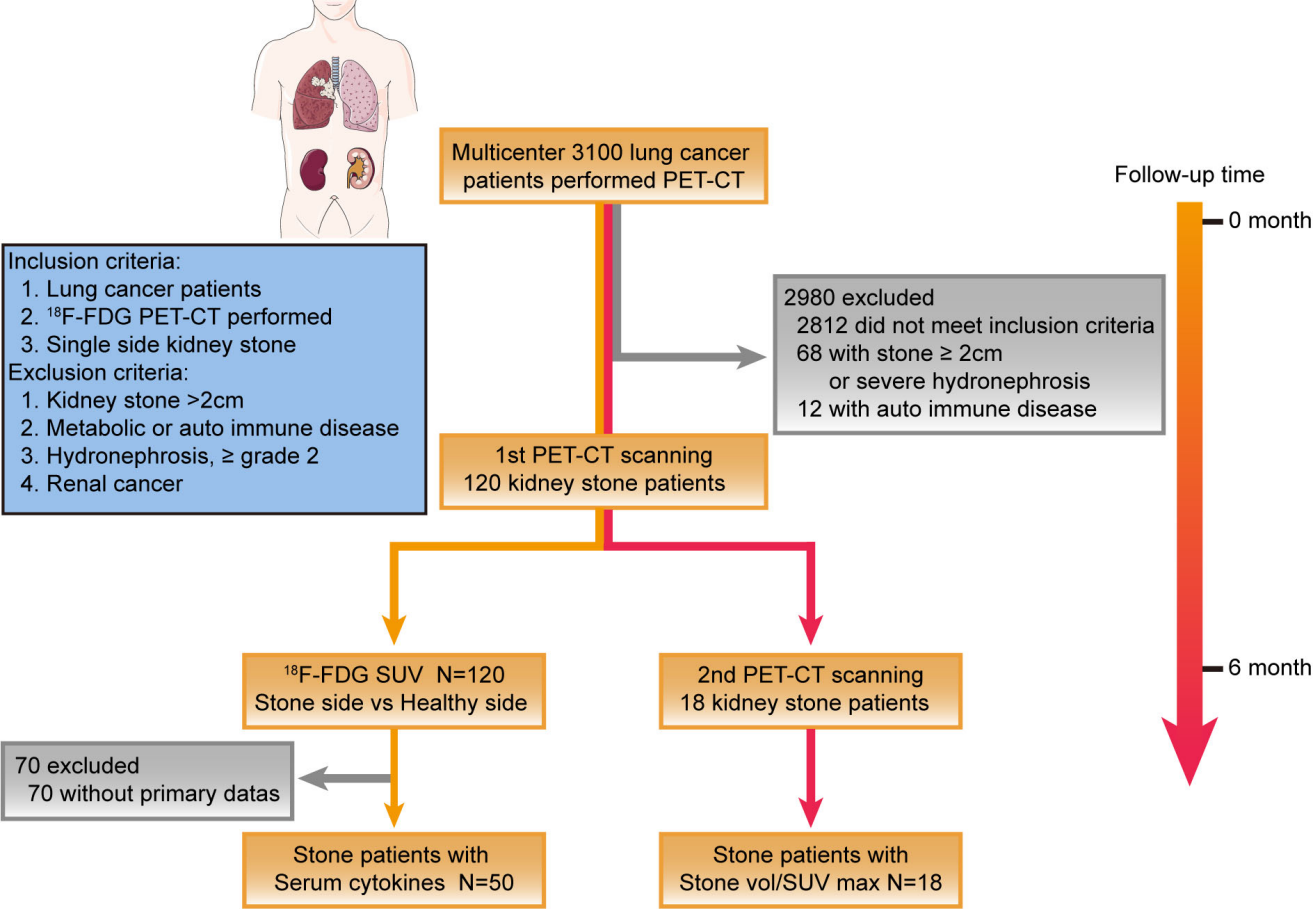
Overview of study protocol and patient inclusion criteria.

From an initial registry of 3,100 lung cancer patients receiving standardized radiotherapy, 120 subjects met stringent inclusion criteria through retrospective screening (**Figure 1**). The cohort demonstrated balanced gender distribution (51.7% male) with median baseline parameters: age 67.3 years, CRP 83.2 mg/L, and leukocytosis 12.6×10^9^/L. Microbiologically confirmed urinary tract infection were identified in 64.2% (n=77), with Enterobacteriaceae predominance (*E. coli* 46.7%, *E. faecalis* 7.5%) (**Table 2**). Diagnostic-quality FDG-PET/CT acquisitions were achieved in 95.8% of cases (115/120), while 4.2% (5/120) required exclusion due to motion artifacts.

**Table 2:** Clinical characteristics of 120 patients diagnosed with kidney stones.

Characteristic	Value
Age (years)	67.3 (32)
Gender	
Male	62 (51.7%)
Female	58 (48.3%)
Kidney stone volume (mm^3^)	1093.2 (968)
CRP (mg/L)	83.2 (63)
WBC count (×10^9^/L)	12.6 (8.1)
Cytokines	
IL-1β (pg/ml)	11.4 (5.8)
TNF-α (pg/ml)	11.9 (4.7)
IL-10 (pg/ml)	26.3 (13.2)
Pathogen from urine culture	
*Escherichia coli*	56 (46.7%)
*Enterococcus faecalis*	9 (7.5%)
*Klebsiella pneumoniae*	5 (4.2%)
*Staphylococcus saprophyticus*	4 (3.3%)
*Enterococcus* spp	3 (2.5%)
Negative culture findings	43 (35.8%)
Quality of PET image	
Good	83 (69.2%)
Reasonable	26 (21.7%)
Poor	11 (9.2%)
^18^F-FDG outcome	
^18^F-FDG positive	76 (63.3%)
^18^F-FDG negative	44 (36.7%)
Ture positive	66 (55.0%)
Ture negative	33 (27.5%)
False positive	10 (8.3%)
False negative	11 (9.2%)

*CRP*, C-reactive protein; *WBC*, white blood cell; *PET*, positron emission tomography; *^18^F-FDG*, ^18^F-Fluorodeoxyglucose.

Analysis of additional clinical variables is presented in **Table 1**. Antibiotic use within 7 days prior to PET/CT was documented in 35.8% of patients, with comparable frequency between PET/CT-positive and negative groups (32.5% vs. 41.9%, *P* = 0.21). Patients with PET/CT-positive findings were significantly more likely to present with fever (53.2% vs. 25.6%, *P* = 0.003) and flank pain (64.9% vs. 41.9%, *P* = 0.01), and less likely to be asymptomatic (11.7% vs. 32.6%, *P* = 0.006). Urinalysis showed significantly higher microscopic white blood cell counts in PET/CT-positive patients (15.8 ± 9.2 vs. 6.3 ± 4.1/HPF, *P* < 0.001). On non-contrast CT, perinephric stranding was more common in PET/CT-positive patients (23.4% vs. 9.3%, *P* = 0.049).

### Diagnostic performance of FDG-PET/CT

Focal renal ^18^F-FDG avidity correlated with infectious foci in 64.2% of studies (77/120). When benchmarked against composite diagnostic standards (discharge diagnosis + urine culture + clinical follow-up), PET/CT demonstrated 11 false-negative and 10 false-positive interpretations. Quantitative accuracy metrics revealed: sensitivity 85.7% (95%CI 75.5-92.3), specificity 76.7% (61.0-87.7), positive predictive value 86.8% (76.7-93.2), negative predictive value 75.0% (59.3-86.3) (**Table 3**).

**Table 3:** Diagnostic performance of FDG PET/CT.

Statistic	Value	95% CI
Sensitivity	85.7%	75.5%-92.3%
Specificity	76.7%	61.0%-87.7%
Positive predictive value	86.8%	76.7%-93.2%
Negative Predictive Value	75.0%	59.3%-86.3%
Positive likelihood ratio	6.6	3.68-11.84
Negative likelihood ratio	0.33	0.20-0.56

*CI*, confidence interval; *PET/CT*, positron emission tomography/computed tomography; *FDG*, fluorodeoxyglucose.

### PET/CT revealed significant high ^18^F-FDG uptake in the renal parenchyma associated with kidney stone

**Figure 2:**
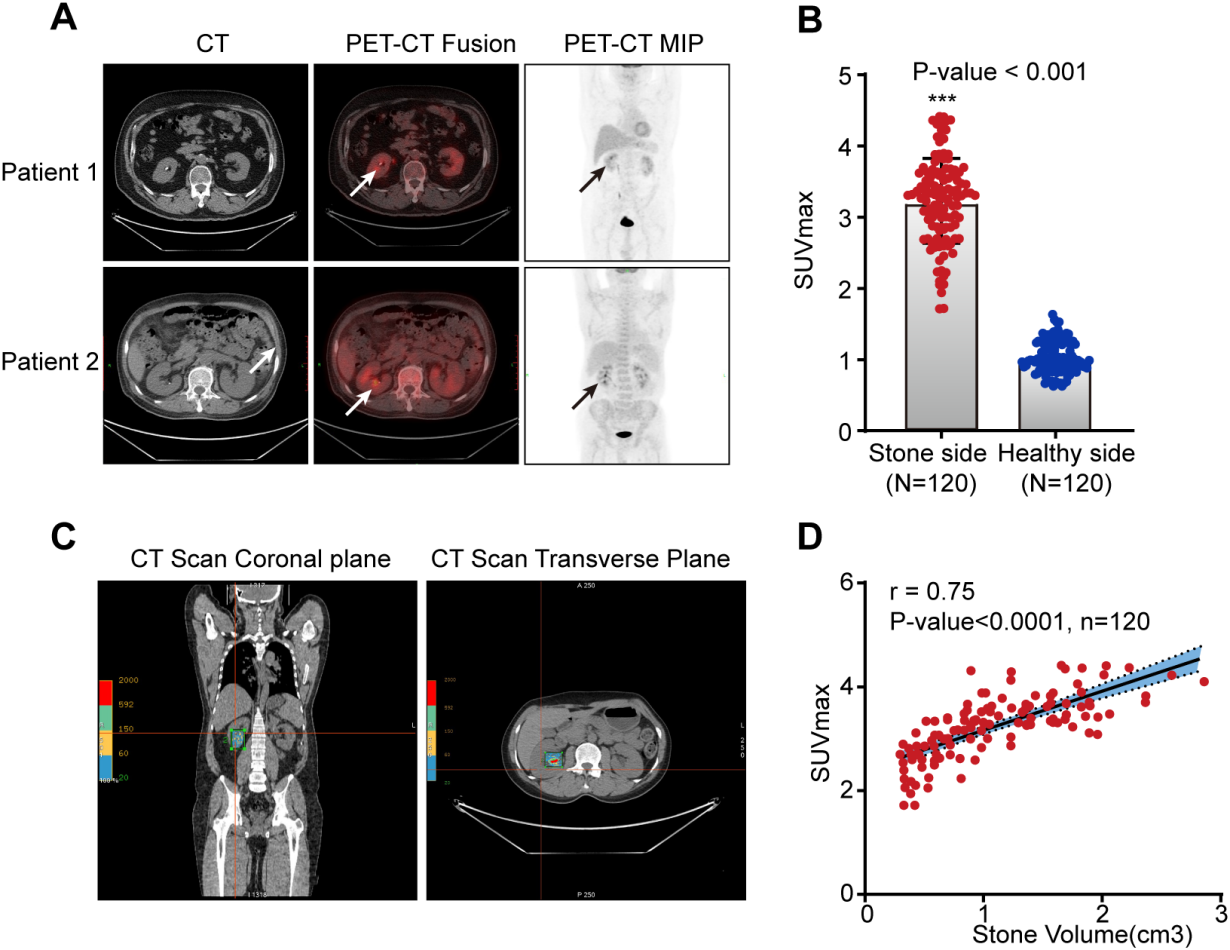
The correlation between stone volume and infection status in the renal parenchyma. **(A)** PET/CT assesses the degree of renal infection in patients with stone. Axial CT, axial fused PET/CT and coronal PET maximum intensity projection (MIP) images measured the uptake of ^18^F-FDG in the kidney. White and black arrows mark kidney stone and ^18^F-FDG accumulation in the renal parenchyma. **(B)** SUVmax value of ^18^F-FDG PET/CT scans on the stone-bearing and healthy sides of kidney stone patients (n = 120). **(C)** Coronal and axial CT imaging show the range of kidney stone. **(D)** Pearson’s correlation coefficient analysis of the ^18^F-FDG SUVmax value and stone volume (n = 120). Data are displayed as the mean ± SD. ***P < 0.001, as determined by Student’s t test.

**Figure 3:**
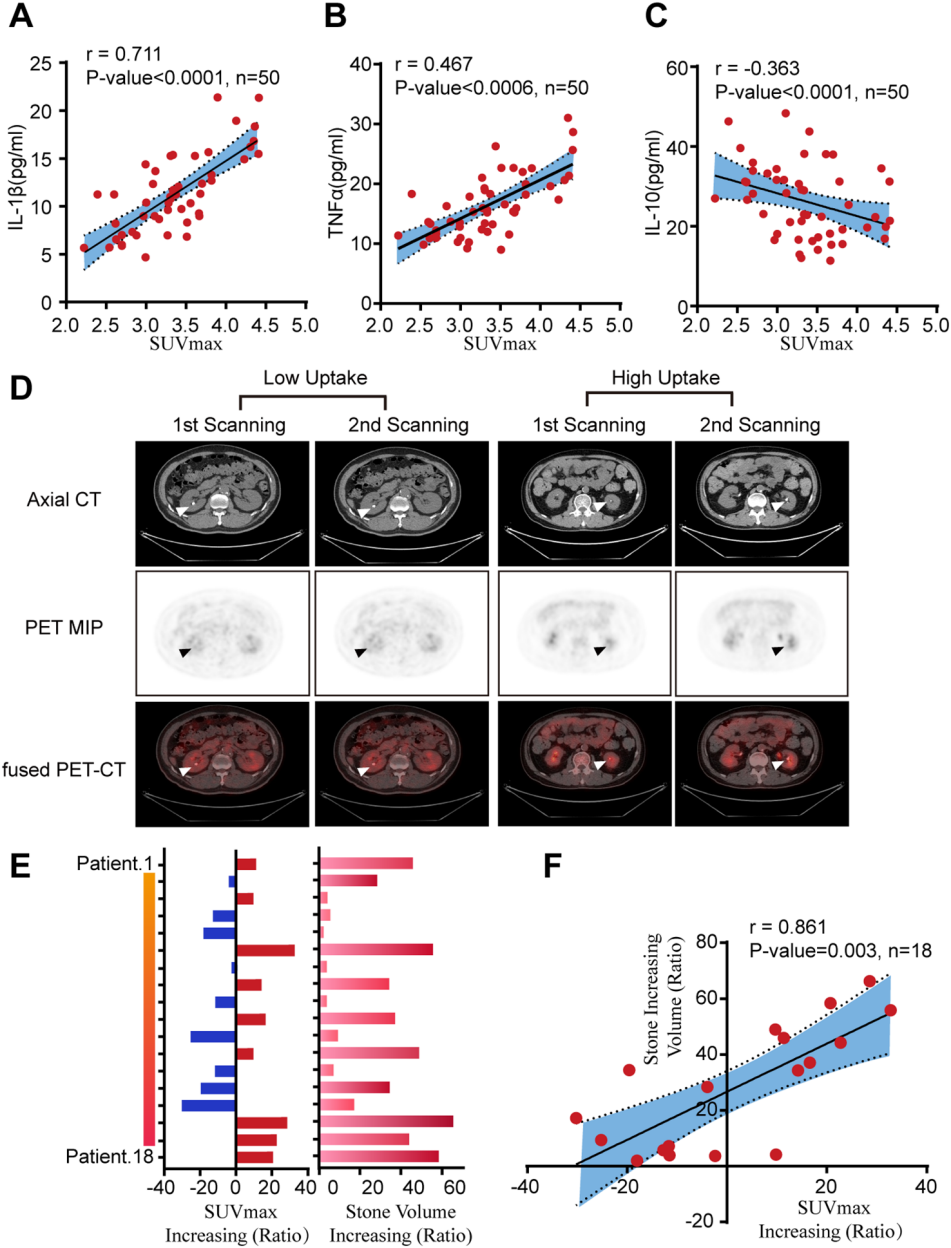
Kidney stone causing renal inflection progression are apparently related to the growth of stone. **(A–C)** Pearson’s correlation test analysis of the SUVmax value in the renal parenchyma and serum IL-1β, TNF-α and IL-10 levels (n = 50). **(D)** Axial CT, axial PET MIP and axial fused PET/CT images measuring renal ^18^F-FDG uptake. **(E)** The increasing ratio of intrarenal SUVmax and stone volume for eighteen who performed twice PET/CT scan after 6 months. **(F)** Pearson’s correlation coefficient analysis of the SUVmax changing ratio in the renal parenchyma and stone volume changing ratio (n = 18).

Based on the large differences in the densities of the renal parenchyma and stone, we used the 3D BOX tool to identify larger than 100-HU area of the kidney to quickly and accurately measure the volume of kidney stone. By comparing the SUVmax in the kidney on the stone side with that in the healthy, non-stone-bearing kidney of these 120 patients, we found that the uptake of ^18^F-FDG in the renal parenchyma on the stone side was obviously higher than that in the healthy kidney in all patients (*P* < 0.001) (**Figures 2A, B**). After three-dimensional reconstruction, the renal stone volume was accurately measured (**Figure 2C**). Moreover, the association between SUVmax in the renal parenchyma and stone size was established by Pearson’s correlation coefficient analysis. We found that the larger the size of the stone was, the higher the ^18^F-FDG uptake in the renal parenchyma, with a positive correlation (r=0.75, *P* < 0.0001) (**Figure 2D**). In addition, the serum cytokines IL-1β, IL-10 and TNF-α, which are commonly used indicators of systemic inflammatory responses in clinical settings, were measured in 50 of these 120 kidney stone patients. The findings showed that in the stone-bearing kidney, with increased uptake of ^18^F-FDG, the level of serum pro-inflammatory cytokines (including IL-1β and TNF-α) increased correspondingly, while the expression of the anti-inflammatory cytokine IL-10 decreased (**Figures 3A–C**). These findings indicate that increased ^18^F-FDG uptake in the renal parenchyma is associated with inflammatory activity in patients with kidney stones and may provide imaging information relevant to clinically suspected renal infection.

Meanwhile, we further analyzed 120 patients with kidney stone who underwent PET/CT examinations at three medical centers, among whom 18 received a second PET/CT scan six months later. A comparison of the two examinations demonstrated that patients with a high SUVmax values in renal parenchyma area had a significantly increased size of their kidney stone at the second occasion (**Figure 3D**). As shown in **Figures 3E, F** and **Supplementary Table 1**, we found that the rate of stone volume growth correlated with the increased ratio of infection of the renal parenchyma as measured between the two scans. In other words, longitudinal changes in renal parenchymal FDG uptake and stone volume appeared to occur in parallel. Within this small follow-up subset, patients exhibiting increased renal FDG uptake typically demonstrated concurrent stone growth, whereas those with decreased uptake showed relative stone volume stability or reduction. These findings suggest a close association between renal inflammatory activity and stone progression.

### The role of PET/CT for the detection of inflammation in the nephrocalcinosis mouse model

**Figure 4:**
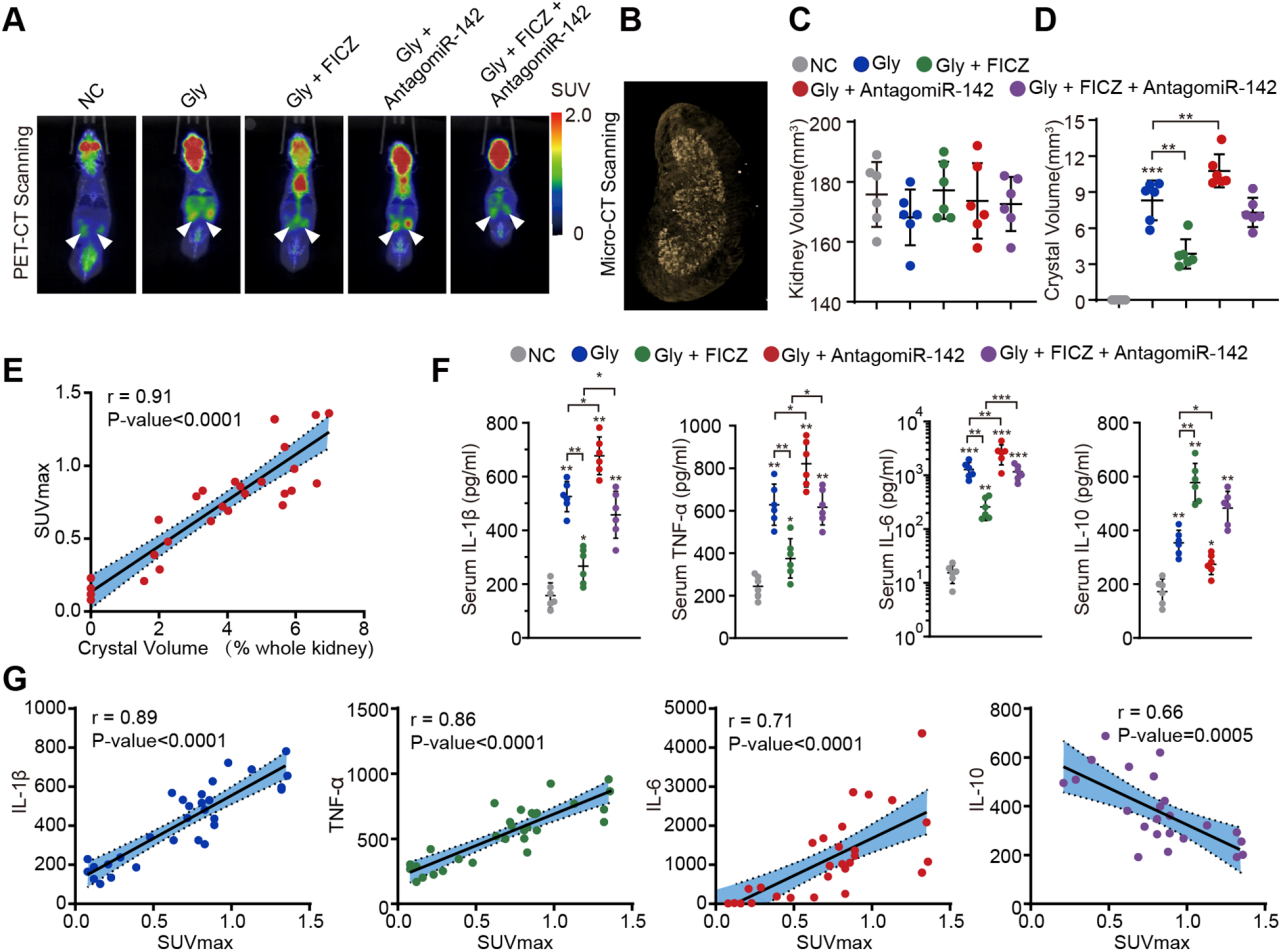
^18^F-FDG micro-PET/CT and micro-CT accurately assess renal inflammation status in the CaOx nephrocalcinosis mouse model. **(A)** Micro-PET/CT shows the ^18^F-FDG uptake situation; white arrows mark ^18^F-FDG accumulation in mouse kidney. **(B–D)** Micro-CT was used to measure the volume of both the mouse kidney and CaOx crystal in the kidney. **(E)** Pearson’s correlation coefficient analysis of the SUVmax and CaOx crystal percentage of the whole kidney (n=30). **(F)** Expression of inflammatory factors in the blood of mice after different treatments. **(G)** Pearson’s correlation test analysis of the levels of inflammatory factors and kidney SUVmax n = 6 per group. Data are displayed as the mean ± SD. *P < 0.05; **P < 0.01, ***P < 0.001, as assessed via one-way ANOVA.

To further investigate CaOx crystal-induced renal inflammation and assess the effects of ^18^F-FDG *in vivo*, micro-PET/CT was applied in our previous study (30). We established a glyoxylate-induced mouse model of CaOx nephrocalcinosis, and mice were treated with FICZ (anti-inflammatory) or antagomiR-142 (pro-inflammatory) administration, followed by the measurement of ^18^F-FDG uptake and local infection level. Micro-PET/CT imaging demonstrated a significantly increased renal uptake of ^18^F-FDG in the glyoxylate-induced CaOx nephrocalcinosis group, whereas FICZ treatment was associated with reduced renal inflammatory activity (**Figure 4A**). In addition, we used micro-CT to accurately display kidney volume and CaOx crystals in the whole kidney after 3D reconstruction (**Supplementary Video S1**; **Figures 4B–D**). Interestingly, the degree of renal inflammation recorded with micro-PET/CT was strongly and positively correlated with the proportion of CaOx crystals in the whole kidney (Stone _Vol_/Kidney _Vol_) (**Figure 4E**). Consistent with the results in stone patients, the degree of renal crystallization was reduced after renal inflammation treated. To further evaluate the diagnostic performance of ^18^F-FDG-PET/CT *in vivo*, pro-inflammatory cytokines (IL-1β, IL-6, and TNF-α) and an anti-inflammatory cytokine (IL-10) were measured to determine systemic inflammation responses after treatments in the CaOx nephrocalcinosis mouse model (**Figure 4F**). The results suggested that after putting renal loci infection (high ^18^F-FDG uptake) under control, the systemic pro-inflammatory cytokines also decreased correspondingly (**Figure 4G**). Importantly, this animal model reflects sterile crystal-induced inflammation rather than proven infection. Therefore, the murine findings should be interpreted as evidence that FDG uptake tracks inflammatory activity associated with CaOx crystal burden but cannot by itself distinguish infection from sterile inflammation. Together, the human and mouse data support PET/CT as a marker of renal inflammatory burden, while definitive discrimination between infection and sterile crystal-driven inflammation still requires integration with microbiology and clinical assessment.

## Discussion

Our findings establish ^18^F-FDG PET/CT as an effective imaging modality for detecting cryptic infectious foci in nephrolithiasis patients with non-diagnostic initial workup. The inclusion of lung cancer patients reflects a pragmatic clinical cohort in which PET/CT imaging is routinely performed, allowing retrospective evaluation of renal metabolic activity without additional imaging exposure. In our patient population, PET/CT exhibited notable detection capability, successfully identifying infective foci in 76 cases (98.7% of culture-positive patients) with diagnostic performance parameters of 85.7% sensitivity and 76.7% specificity relative to composite clinical reference standards.

Importantly, the false-positive and false-negative findings observed in this study are biologically plausible. False-positive scans may result from sterile inflammation induced by CaOx crystals, as supported by our murine model showing FDG uptake correlated with crystal burden without infection. Additional contributors include recent antibiotic use, low-grade obstructive inflammation, or urinary tracer interference. Conversely, false-negative results may occur when infection is focal, low-grade, or partially treated. Clinical data provide further context: antibiotic use trended longer in PET/CT-negative patients (5.7 vs. 4.8 days, *P* = 0.15), suggesting partial treatment may attenuate FDG avidity. Notably, PET/CT detected uptake in 11.7% of asymptomatic patients, highlighting its ability to identify clinically occult infections. Typical symptoms (fever, pain) and microscopic pyuria were strongly associated with PET/CT positivity (*P* < 0.01 and *P* < 0.001), while hematuria was not, indicating FDG uptake reflects inflammation rather than mechanical irritation. Among CT features, perinephric stranding was significantly more common in PET/CT-positive patients (23.4% vs. 9.3%, *P* = 0.049), suggesting metabolic activity adds independent information beyond obstruction. Taken together, these findings indicate that renal FDG uptake should not be interpreted in isolation; rather, PET/CT is best viewed as a complementary biomarker that must be integrated with urine culture, inflammatory indices, anatomical imaging, and clinical follow-up to distinguish true infection from sterile inflammation.

While contrast-enhanced CT remains the standard for anatomical assessment of nephrolithiasis, its sensitivity for detecting renal parenchymal infection is limited, particularly in the absence of abscess formation or pyonephrosis. In a study by Soulen et al., contrast-enhanced CT had a sensitivity of only 67% for acute pyelonephritis compared to histopathology (31). More recent studies have reported higher sensitivity (81.0%) (32), but CT still may fail to detect early or low-grade infections. Compared with conventional imaging methods, ^18^F-FDG PET/CT offers distinct advantages in early lesion detection, diagnostic accuracy, and image resolution. In addition, it provides particular benefits for elderly and immunocompromised patients due to its convenience and ease of clinical application (33). Recently, studies on ^18^F-FDG PET/CT to assess acute kidney injury and chronic kidney inflammation have increased (34–37). However, little is known about the detection and evaluation of renal infection and damage caused by kidney stone or CaOx crystals.

PCNL is currently the first-line minimally invasive treatment for kidney stones, particularly recommended for managing large stone (1, 38, 39). However, complications including extravasation, fever, and septicemia are important factors affecting the safety of PCNL (40–42). For patients with tumor complicated with nephrolithiasis, if the local infection is not determined before operation, fatal pyemia or septicemia may occur intraoperative and endanger the patient’s life due to their weak immunity (43, 44). For patients with simple kidney stones, obtaining information on local infection and pus accumulation before surgery is beneficial to relieve the pain of and reduce the length of hospital stay. Most importantly, blood and urine tests are currently the primary means for assessing patient’s infection status, yet localized infections caused by stones are often undetectable (45). Research indicates that some patients succumb to urosepsis even with appropriate antibiotic treatment (46). Therefore, understanding the local infection of patients before operation can help clinicians to design personalized treatment therapies and reduce the burden of patients. Prophylactic antibiotics are not required if no significant infection is detected on preoperative PET/CT. When infection is detected, standard anti-infective therapy is administered according to the severity of infection to avoid exacerbation. Furthermore, PET/CT can accurately locate the infected lesions allow operators to prepare for intraoperative suction and drainage in advance, providing a safe operating environment for surgeons. The mammalian kidney is a complex energy factory that meets the high energy demand and has the mitochondrial content only second to that of the heart. The necessary fuel is mainly fatty acid β-oxidation and glycolysis (47, 48). Metabolic reprogramming reflects renal tubular cell injury, representing a compensatory shift toward glycolysis to generate ATP following renal damage (49). This metabolic reprogramming changed the main energy source for the kidneys from fatty acids to glucose. This might be the reason why PET/CT shows high glucose uptake in kidney stone patients as well as in CaOx nephrocalcinosis in mouse kidneys. To the best of our knowledge, we have demonstrated for the first time the potential application value of ^18^F-FDG PET/CT for the evaluation of patients with renal infection caused by kidney stone or CaOx crystals. However, because FDG uptake reflects inflammation rather than infection specificity, PET/CT findings should be interpreted together with microbiological and clinical data when inferring true renal infection.

Some limitations of the current research project should be noted. Firstly, the retrospective design and the specific cohort of lung cancer patients introduce selection bias and limit generalizability to the general nephrolithiasis population. Key clinical variables, such as precise antibiotic history, symptom duration, and detailed urinalysis, were not consistently available; these could have influenced the accuracy of the reference standard. Secondly, whether ^18^F-FDG PET/CT imaging should be carried out was dependent on the clinician’s judgment based on the specific clinical situation. This might have led to selection bias. Nevertheless, with the promotion of disease prevention and the expansion of medical insurance coverage in various countries, clinicians will be more active in arranging PET/CT examination according to the actual situation of patients. Thirdly, regarding radiation safety, the effective dose from a typical ¹^18^F-FDG PET/CT scan ranges from 10 to 25 mSv, which is comparable to or slightly higher than that of a contrast-enhanced abdominal CT (approximately 8–20 mSv). However, modern PET/CT systems and dose-reduction protocols (e.g., time-of-flight, iterative reconstruction) can lower exposure without compromising image quality. In addition, although ^18^F-FDG PET/CT first might have been considered a “high-cost, high-tech” imaging technology, PET imaging now has become a clinical reality. In addition, low-cost methods, such as incorporation of a gamma camera to detect FDG uptake, are also available to patients. The results thus far indicated that FDG is extremely promising and practical for clinical promotion. Furthermore, our exclusion of patients with large (>2 cm) or ureteral stones limits the generalizability of our findings. In these populations, impaired radiotracer excretion due to obstruction may reduce the specificity of PET/CT for detecting parenchymal infection. Future prospective studies are warranted to evaluate the diagnostic performance of PET/CT in such complex stone disease. Despite the limitations listed above, it is beyond doubt that PET/CT will receive widespread application in the future, and we assume that our findings will contribute to stimulating increased research interest in this field.

In conclusion, this study demonstrates that in lung cancer patients with concomitant nephrolithiasis, ¹^18^F-FDG PET/CT is a feasible imaging modality for detecting stone-related renal infection, with sensitivity and specificity of 85.7% and 76.7%, respectively. The correlation between metabolic activity, stone burden, and inflammatory markers supports its potential for preoperative risk assessment in this specific population. However, given the overlap between infection and sterile crystal-induced inflammation, PET/CT findings should always be interpreted alongside microbiological and clinical data. Further prospective studies in unselected nephrolithiasis populations are warranted to validate these findings and establish broader clinical utility.

## Data Availability

The original contributions presented in the study are included in the article/**Supplementary Material**. Further inquiries can be directed to the corresponding authors.

## Supplementary material

Supplementary Table 1 PET/CT examination reveals relationship between intrarenal ^18^F-FDG uptake and kidney stone size.

Supplementary Video 1 Micro-CT 3D reconstruction of CaOx crystal deposits in the whole mouse kidney.
