# Supplementary material for: Evaluation of kidney stone–related renal infection status by 18F-FDG PET/CT in lung cancer patients with concomitant nephrolithiasis: a multicenter study
Source: Front Immunol. 2026 May 19;17:1744163. doi: 10.3389/fimmu.2026.1744163 (PMC13226474; doi:10.3389/fimmu.2026.1744163)
Supplement: Supplementary Table 1 — PET/CT examination reveals relationship between intrarenal 18F-FDG uptake and kidney stone size. [file Table1.docx]

**Supplementary Table S1 PET/CT examination reveals relationship between intrarenal ^18^F-FDG uptake and kidney stone size**

| **Patients** | **SUVmax**  **(stone kidney)** | | **Stone Volume (mm^3^)** | | | **Increasing Ratio (%)** | |
| --- | --- | --- | --- | --- | --- | --- | --- |
|  | **1st Scanning** | **2nd Scanning** | | **1st Scanning** | **2nd Scanning** | **SUVmax** | **Stone Volume** |
| Patient NO.1 | 4.69 | 5.22 | 683.4 | | 997.2 | 11.37% | 45.92% |
| Patient NO.2 | 5.13 | 4.93 | 536.7 | | 689.1 | -3.93% | 28.40% |
| Patient NO.3 | 2.85 | 3.13 | 508.3 | | 529.3 | 9.79% | 4.13% |
| Patient NO.4 | 5.73 | 5.00 | 683.8 | | 723.1 | -12.71% | 5.75% |
| Patient NO.5 | 5.24 | 4.30 | 896.3 | | 913.6 | -17.99% | 1.93% |
| Patient NO.6 | 2.85 | 3.79 | 489.7 | | 763.1 | 32.80% | 55.83% |
| Patient NO.7 | 3.30 | 3.22 | 672.9 | | 697.3 | -2.36% | 3.63% |
| Patient NO.8 | 4.02 | 4.59 | 537.1 | | 721.6 | 14.24% | 34.35% |
| Patient NO.9 | 4.46 | 3.95 | 581.2 | | 603.8 | -11.52% | 3.89% |
| Patient NO.10 | 3.49 | 4.07 | 483.6 | | 662.8 | 16.57% | 37.06% |
| Patient NO.11 | 4.36 | 3.26 | 1832 | | 2003 | -25.23% | 9.33% |
| Patient NO.12 | 4.12 | 4.52 | 1233 | | 1836 | 9.71% | 48.91% |
| Patient NO.13 | 3.69 | 3.26 | 2366 | | 2536 | -11.65% | 7.19% |
| Patient NO.14 | 3.58 | 2.88 | 1033 | | 1389 | -19.55% | 34.46% |
| Patient NO.15 | 3.28 | 2.29 | 1050 | | 1231 | -30.18% | 17.24% |
| Patient NO.16 | 3.15 | 4.05 | 982 | | 1633 | 28.57% | 66.29% |
| Patient NO.17 | 2.59 | 3.18 | 389 | | 561 | 22.78% | 44.22% |
| Patient NO.18 | 2.22 | 2.68 | 332 | | 653 | 20.72% | 58.43% |
| *PET/CT* positron emission tomography/computed tomography, *SUV* standardized uptake values | | | | | | | |
