# Supplementary figures and images for: Evaluation of kidney stone–related renal infection status by 18F-FDG PET/CT in lung cancer patients with concomitant nephrolithiasis: a multicenter study
Source: Front Immunol. 2026 May 19;17:1744163. doi: 10.3389/fimmu.2026.1744163 (PMC13226474; doi:10.3389/fimmu.2026.1744163)

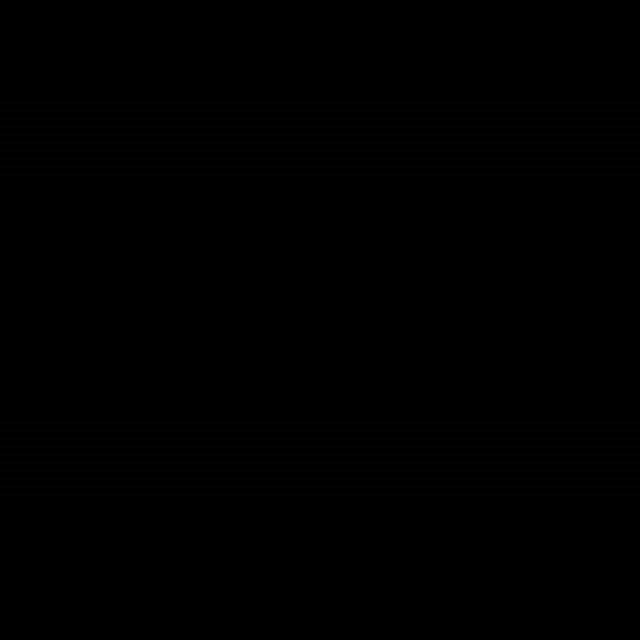

Supplement: Supplementary Video 1 — Micro-CT 3D reconstruction of CaOx crystal deposits in the whole mouse kidney. [file DataSheet1.zip › PET-CT Video 3D reconstruction.gif]
